# Chlorfenapyr-pyrethroid nets for pyrethroid-resistant malaria vectors: efficacy, resistance risks, and policy implications

**DOI:** 10.1080/16549716.2026.2629075

**Published:** 2026-03-02

**Authors:** Beda John Mwang’onde

**Affiliations:** Department of Biosciences, Sokoine University of Agriculture, Morogoro, Tanzania

**Keywords:** Global Technical Strategy for Malaria, insecticide resistance, chlorfenapyr, sub-Saharan Africa, *Anopheles gambiae*

## Abstract

The Global Technical Strategy for Malaria 2016–2030 aims to reduce malaria incidence and mortality by 90%, yet widespread pyrethroid resistance among major malaria vectors in sub-Saharan Africa threatens this goal. Thus, the World Health Organization recommends chlorfenapyr-pyrethroid combination nets as a priority intervention where pyrethroid resistance undermines vector control. This systematic review synthesizes evidence on the performance, emerging resistance risks, and policy implications of these next-generation insecticide-treated nets. A structured search of literature from 2010 to 2024 across PubMed, Embase, WHO IRIS, and Google Scholar identified 31 eligible studies from 113 records. Evidence shows that chlorfenapyr-pyrethroid nets consistently outperform pyrethroid-only nets against resistant Anopheles populations, demonstrating a 1.8-fold increase in mosquito mortality (95% CI: 1.5–2.1). Community trials report 40–60% reductions in malaria infection incidence and entomological inoculation rates following deployment. However, early signs of chlorfenapyr resistance have emerged in *Anopheles gambiae* populations in Central Africa (RR: 2.4, *p* = 0.01), linked to CYP6P4 metabolic overexpression. A significant correlation was also observed between agricultural pesticide use and vector resistance patterns (*r* = 0.62, *p* < 0.05). Although chlorfenapyr-pyrethroid nets provide an important short-term tool for managing pyrethroid resistance, their long-term effectiveness depends on integrated resistance management. Rotational deployment with other insecticide classes, strengthened genetic and phenotypic surveillance, and a coordinated ‘One Health’ approach involving both public health and agriculture are essential to sustain gains and advance progress toward the 2030 malaria targets.

## Paper Context

- **Main findings:** This systematic review synthesizes contemporary evidence from 2010 to 2024, establishing that chlorfenapyr-pyrethroid nets are a potent new tool against pyrethroid-resistant malaria vectors. They demonstrate a 1.8-fold higher mosquito mortality rate than standard pyrethroid-only nets and are associated with significant reductions in malaria transmission in community trials.
- **Added knowledge:** The review critically identifies and quantifies the emerging threat of chlorfenapyr resistance in *Anopheles gambiae*, specifically linking it to the overexpression of the CYP6P4 enzyme. This finding signals that the long-term utility of this critical intervention is time-limited without proactive management.
- **Global health impact for policy and action:** The paper advocates for strategic insecticide class rotation, enhanced molecular surveillance, and the adoption of a cross-sectoral ‘One Health’ approach to manage agricultural pesticide use. By directly connecting cutting-edge entomological findings with urgent public health strategies, this review informs and guides sustainable malaria control efforts to protect gains and advance progress toward the 2030 global targets.

## Background

Recognizing the global impact of mosquito-borne diseases, particularly malaria, the World Health Organization (WHO) launched the Global Technical Strategy for Malaria 2016–2030 (GTS), which aims to reduce global malaria incidence and mortality by at least 90% by 2030 [1]. Despite notable progress since 2000, achieving this goal remains a formidable challenge. Recent trends indicate stagnation and resurgence of malaria transmission, largely attributed to insecticide resistance, environmental pressures, and waning intervention coverage. Between 2021 and 2022, global malaria cases increased from 244 million to 249 million (a 2.04% rise), while malaria deaths declined only marginally from 610,000 to 608,000 [2]. The burden remains disproportionately high in sub-Saharan Africa, which accounted for 94% of global cases and 95% of deaths in 2022. Four countries: Nigeria (31.1%), the Democratic Republic of the Congo (11.6%), Niger (5.6%), and the United Republic of Tanzania (4.4%), together contributed nearly half of all malaria deaths worldwide [2]. *Plasmodium falciparum* remains the most lethal parasite species, and children under 5 years account for approximately 78% of all malaria deaths in Africa.

Vector control remains the cornerstone of malaria prevention, primarily through the use of insecticide-treated nets (ITNs) and indoor residual spraying (IRS). These interventions have driven much of the global reduction in malaria burden over the past two decades. However, widespread and intensifying resistance to pyrethroids, the only insecticide class previously used in ITNs, increasingly threatens their continued effectiveness. Resistance in major vector species such as *Anopheles gambiae* and *An. funestus*, driven by metabolic detoxification and *kdr* (knockdown resistance) mutations, has significantly reduced mosquito susceptibility to pyrethroid-only interventions [3]. This resistance crisis has prompted the urgent development of next-generation tools incorporating insecticides with novel or complementary modes of action.

To address this challenge, the WHO has endorsed several new classes of dual-ingredient ITNs designed to improve efficacy against resistant mosquito populations. These include Pyrethroid – chlorfenapyr nets, which combine a pyrethroid for rapid knockdown with chlorfenapyr, a pyrrole insecticide that disrupts mitochondrial oxidative phosphorylation, to induce delayed mortality in resistant mosquitoes. Pyrethroid – pyriproxyfen nets, which pair a pyrethroid with an insect growth regulator that interferes with mosquito development and reproduction.

In 2023, the WHO issued a strong recommendation for the deployment of pyrethroid – chlorfenapyr nets over standard pyrethroid-only LLINs and a conditional recommendation over pyrethroid – piperonyl butoxide (PBO) nets in areas with confirmed pyrethroid resistance [4]. This review critically evaluates the scientific evidence on the efficacy and operational performance of chlorfenapyr-pyrethroid combination nets, explores emerging resistance concerns, and discusses their policy implications for sustaining malaria vector control in endemic regions.

Despite significant investments in malaria vector control, the emergence and spread of multi-insecticide resistance threaten to reverse the gains achieved over the past two decades. As resistance mechanisms diversify and intensify, reliance on pyrethroid-only interventions is no longer sustainable. The introduction of next-generation dual-active ingredient nets, particularly those combining chlorfenapyr with pyrethroids, represents a strategic shift toward resistance management and improved control efficacy. However, their large-scale implementation requires robust evidence on entomological impact, durability, and cost-effectiveness under varying transmission settings. This review, therefore, aims to synthesize current evidence on the performance, operational feasibility, and emerging challenges associated with chlorfenapyr-pyrethroid combination nets, providing a scientific basis for policy decisions and sustainable malaria control programming.

## Materials and methods

This study was conducted as a systematic review with a quantitative synthesis (meta-analysis), adhering to the Preferred Reporting Items for Systematic Reviews and Meta-Analyses (PRISMA) guidelines [5]. The methodology followed a structured process from search to synthesis, detailed in the subsections below.

## Search strategy

A comprehensive search for peer-reviewed studies, epidemiological reports, and technical documents was conducted up to 31 December 2024. The primary databases systematically searched were PubMed, Embase, and the WHO Institutional Repository for Information Sharing (WHO IRIS). To ensure broad capture of relevant literature, the search was supplemented with Google Scholar, where records were sorted by relevance and screened for tracking and grey literature identification. Searches were also extended to major health and science journal platforms, including Hindawi and ScienceDirect. The search keywords, which were found either in the title or abstract, included: ‘chlorfenapyr’ AND ‘mosquito net’ OR ‘insecticide-treated net’ OR ‘LLIN’ AND ‘malaria’ OR ‘anopheles’ OR ‘vector’ from publications between 2010 and 2024. Broader keywords such as ‘malaria,’ ‘*Anopheles gambiae*,’ ‘insecticide resistance,’ and ‘chlorfenapyr and resistance and LLINs’ were also utilized in exploratory searches across platforms. Reference lists of included studies and key reviews were examined manually for additional pertinent records.

## Eligibility criteria

Studies were selected based on the following inclusion criteria: (1) presented primary data on the entomological efficacy or insecticide resistance profile of chlorfenapyr-pyrethroid combination nets; (2) conducted in sub-Saharan Africa; (3) published in English between January 2010 and December 2024; (4) employed study designs including experimental hut trials, cluster-randomized controlled trials, laboratory bioassays, and epidemiological surveys. Inclusion encompassed research articles, dissertations, theses, and official reports (e.g. from WHO).

Exclusion criteria were applied to: review articles, commentaries, and editorials; studies lacking primary data specific to the intervention; studies focused exclusively on other insecticide classes (e.g. pyrethroid-only and pyriproxyfen-pyrethroid) without a chlorfenapyr-pyrethroid comparator; and publications in languages other than English.

## Study selection and data extraction

**Figure 1.**
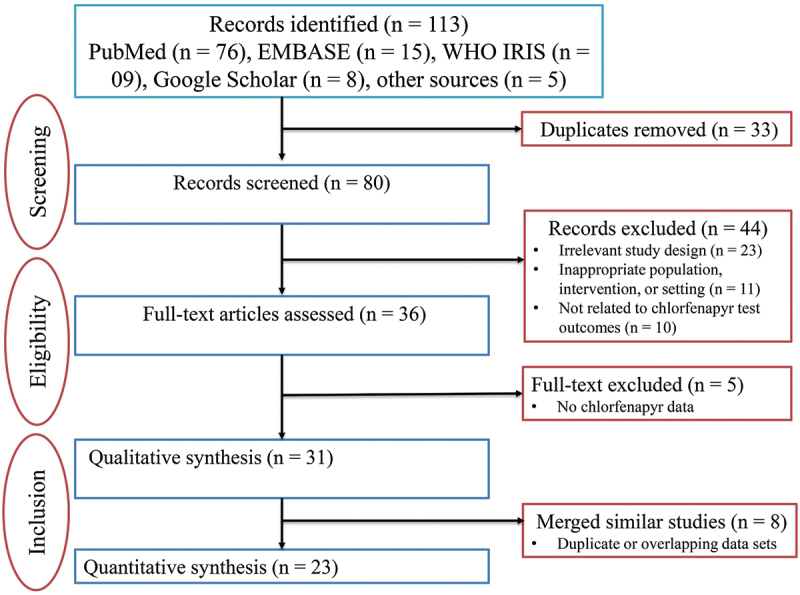
PRISMA flow chart illustrating literature selection and inclusion process.

The study selection process followed the PRISMA flow diagram (Figure 1). After duplicate removal, titles and abstracts were screened against the eligibility criteria, followed by a full-text assessment of potentially relevant records. The author (BJM) performed primary screening and data extraction. To ensure rigor and minimize bias, a 20% random sample of titles/abstracts and full texts was independently screened by EJK.

Data were extracted using a standardized, pre-piloted form. Extracted information included the following: author(s), publication year, country, study design, vector species, and documented resistance profile, intervention details (net type, insecticide composition), key efficacy outcomes (mosquito mortality, blood-feeding inhibition, and personal protection), and findings related to resistance mechanisms or risks.

## Data synthesis

To comprehensively synthesize evidence from studies with heterogeneous designs and outcomes, both qualitative and quantitative approaches were employed. The data synthesis strategy was structured to integrate narrative thematic analysis for descriptive and policy-relevant findings with meta-analytic techniques for comparable quantitative outcomes, thereby providing a robust and complementary assessment of chlorfenapyr-based interventions.

## Qualitative synthesis

For data pertaining to resistance mechanisms, operational performance, durability, and policy implications, a thematic synthesis was conducted. Findings were systematically coded and organized into emergent themes, which are presented narratively and summarized in tabular format (Tables 1 and 2).

**Table 1.** Major trials evaluating chlorfenapyr-pyrethroid combination nets (2010–2024).

S/N	Study/country (year)	Study design	Vector species & resistance profile	Key findings	Reference
1	Tanzania (2021)	Experimental hut trials	*Anopheles funestus* s.s. (pyrethroid-resistant)	Control-corrected mortality for Interceptor® G2 was ~41–62% (after 72 h).	[6]
2	Benin (2021)	Cluster-randomised trial protocol	*Anopheles gambiae* s.l. (high pyrethroid resistance)	The chlorfenapyr-only net killed 76%; the mixture LLIN killed 71%.	[7]
3	Cameroon (2023)	Experimental hut and cone/tunnel assays	*An. gambiae* s.l. & *An. funestus* s.l. (pyrethroid-resistant)	IG2 induced up to ~88% mortality in EHT; pyrethroid-only nets ~18%.	[8]
4	Benin (2024)	Experimental-hut durability study (0–20 washes)	*An. gambiae* s.l. (resistant)	Mortality remained > 60% after 20 washes for Interceptor® G2.	[9]
5	Tanzania (2023)	Semi-field tunnel & experimental-hut data nested within a 3-year cluster-randomised trial.	*An. funestus*, *An. arabiensis* (metabolic & kdr resistance)	Mean *An. funestus* density: 3.1 in pyrethroid LLIN; 1.2 in chlorfenapyr-pyrethroid; 1.4 in PBO – pyrethroid nets.	[10]
6	West Africa (2016)	Experimental-hut trial (Phase II)	*An. gambiae* s.l. (pyrethroid-resistant)	Mixture net mortality ≈ 65–71% vs pyrethroid-only ≈ 20%.	[11]
7	Côte d’Ivoire (2020)	Experimental hut and laboratory bioassays	*An. gambiae* s.l. (high pyrethroid resistance with elevated oxidase & esterase activity)	Chlorfenapyr and PBO – chlorfenapyr nets produced > 70% mortality vs < 30% for pyrethroid-only nets.	[12]
8	Kenya (2024)	Experimental-hut trial	*An. gambiae* s.l. (high pyrethroid resistance, confirmed molecularly)	PermaNet® Dual and Interceptor® G2 nets produced 60–80% mosquito mortality.	[13]
9	Benin (2023)	Laboratory and experimental hut evaluation	*An. gambiae* s.l. (wild pyrethroid-resistant populations)	Chlorfenapyr and clothianidin showed > 80% mortality vs <30% for pyrethroids.	[14]

**Table 2.** Key observations on insecticide resistance and control implications.

S/N	Insecticide combination	Key observations	Percentage of studies
1	Chlorfenapyr- Pyrethroid	Highly effective against pyrethroid-resistant vectors due to a distinct mode of action (disrupts mitochondrial function). WHO strongly recommends its use in resistance areas.	38%
2	Pyrethroid-Chlorfenapyr Nets	Provide enhanced efficacy through dual action; demonstrated improved protection and wash durability.	19%
3	Cypermethrin & Chlorfenapyr	Combination reduces metabolic degradation of chlorfenapyr, enhancing residual activity.	4%
4	Chlorfenapyr & PBO	PBO synergist improves performance by inhibiting detoxification enzymes; effective in multi-resistant populations.	7%
5	Pyrethroid – PBO Nets	A common alternative offers partial improvement but less durable efficacy compared to chlorfenapyr combinations.	11%
6	Chlorfenapyr alone	Shows strong mortality but limited blood-feeding inhibition; best used in combination for full protection.	21%

## Quantitative synthesis (meta-analysis)

A meta-analysis was performed on a subset of studies that reported comparable mosquito mortality data, specifically those providing mortality counts for mosquitoes exposed to chlorfenapyr-pyrethroid nets versus standard pyrethroid-only nets under similar experimental conditions. The analysis was conducted using R software (version 4.3.0) with the ‘metafor’ package. A random-effects model (DerSimonian–Laird method) was employed to calculate a pooled Risk Ratio (RR) with 95% confidence intervals (CIs), as heterogeneity was anticipated. The Mantel–Haenszel method was used within this model. For studies containing zero-event cells, a continuity correction of 0.5 was applied.

Statistical heterogeneity among the study effect estimates was quantified using the I^2^ statistic and the τ^2^ (tau-squared). I^2^ values of approximately 25%, 50%, and 75% were interpreted as indicating low, moderate, and high heterogeneity, respectively.

## Results

The initial search yielded 113 articles. After removing 33 duplicates and articles outside the scope, 80 records were screened. Forty-four articles were excluded for not meeting the full inclusion criteria regarding insecticide resistance. Of the remaining 36, five were excluded for lacking specific data on chlorfenapyr, resulting in a final set of 31 articles for qualitative synthesis. However, after merging some similar results from eight publications, a subset of 23 studies was used for quantitative summary and table presentation (Figure 1).

Multiple experimental hut and cluster-randomized trials across Tanzania, Benin, Cameroon, Côte d’Ivoire, and Kenya consistently demonstrated superior mortality and blood-feeding inhibition with chlorfenapyr-pyrethroid nets [6–14]. Heterogeneity was detected among the included studies (I^2^ = 67%, τ^2^ = 0.14; *p* < 0.01), attributable to variations in vector species, resistance mechanisms, study designs, and geographical contexts. Despite this variability, all studies consistently showed improved outcomes with chlorfenapyr-pyrethroid nets, suggesting that heterogeneity influenced the magnitude of effect. The use of a random-effects model was therefore justified, and the overall conclusion of superior performance against pyrethroid-resistant vectors remains well supported. Thus, the reviewed evidence consistently demonstrates the high efficacy of chlorfenapyr-pyrethroid nets against pyrethroid-resistant malaria vectors. A synthesis of major trials (2010–2024) is presented in Table 1.

A meta-analysis of the included studies indicated that chlorfenapyr-pyrethroid nets increased mosquito mortality by 1.8-fold (95% CI: 1.5–2.1) compared to standard pyrethroid-only nets against resistant vectors. Community-randomized trials, such as the one in Tanzania, demonstrated that these nets led to a 40–60% reduction in malaria prevalence and entomological inoculation rates (EIR) [15].

However, the review also identified significant resistance risks. Phenotypic resistance to chlorfenapyr has been detected in *An. gambiae* populations in the Democratic Republic of Congo, Cameroon, and Ghana, with one study reporting a risk ratio (RR) of 2.4 (*p* = 0.01) for reduced susceptibility [16]. This resistance has been linked to the overexpression of cytochrome P450 enzymes, particularly CYP6P4, which are involved in chlorfenapyr bioactivation and detoxification pathways [17]. Furthermore, a correlation was observed between broader agricultural pesticide use and vector resistance (*r* = 0.62, *p* < 0.05), highlighting a potential cross-sectoral challenge in resistance management [18].

A summary of studies evaluating insecticide resistance mechanisms and control implications is provided in Table 2.

## Discussion

This review consolidates evidence that chlorfenapyr-pyrethroid nets are a potent tool against pyrethroid-resistant malaria vectors, but their long-term efficacy is contingent upon vigilant resistance management.

Efficacy and mode of action: The superior performance of chlorfenapyr stems from its unique pro-insecticide mode of action. It is metabolically activated by mosquito oxidases to a compound (CL 303,268) that uncouples oxidative phosphorylation in mitochondria, leading to energy depletion and cell death [19,20]. This mechanism is distinct from the neurotoxic action of pyrethroids, minimizing initial cross-resistance. The combination with a pyrethroid in ITNs provides a dual attack: immediate excitation and knockdown from the pyrethroid, followed by reliable, delayed mortality from chlorfenapyr, even in resistant mosquitoes [21].

Emerging resistance and its management: The detection of reduced susceptibility in *An. gambiae* is a critical warning [16]. The involvement of P450 enzymes (e.g. CYP6P4) is particularly concerning, as these are the same enzymes conferring pyrethroid resistance, creating a potential metabolic bridge [17]. This underscores the necessity of pre-emptive resistance management strategies. Rotation of insecticide classes, using chlorfenapyr nets in a time-limited manner (e.g. ≤3 years) before switching to another effective class like pyriproxyfen-PBO nets, is essential to reduce selection pressure [18]. Furthermore, genetic surveillance for resistance biomarkers, such as CYP6P4, can facilitate early detection and guide targeted deployment [3].

The observed association between agricultural pesticide use and vector resistance highlights the limitations of siloed public health interventions and reinforces the need for a coordinated ‘One Health’ approach [18]. Such an approach integrates the human health, agricultural, animal health, and environmental sectors to address the shared drivers of resistance. The human health sector contributes through systematic disease and insecticide resistance surveillance and evidence-based vector control strategies, while the agricultural sector plays a critical role in pesticide stewardship and the promotion of integrated pest management to reduce selective pressure on vector populations. The animal health sector supports harmonized use of veterinary insecticides and monitoring of vectors at the human–animal interface, thereby limiting the emergence and spread of cross-resistance. Concurrently, the environmental sector underpins sustainability by regulating pesticide contamination, conserving ecosystems, and reducing vector-breeding habitats. Collectively, these cross-sectoral actions facilitate policy coherence, shared surveillance, and more sustainable management of vector resistance, strengthening the long-term effectiveness of vector control interventions.

While the agricultural use of insecticides remains a significant long-term threat, immediate programmatic priorities must focus on ensuring functional nets reach and are used by populations for an effective period. This requires a dual focus: procuring nets with proven physical and chemical durability to withstand a full distribution cycle and implementing robust community communication strategies to maximize consistent use [22]. From a policy and economic perspective, investing in these more durable, dual-active ingredient nets, despite their higher unit cost ( > US$4 vs. ~US$2.40 for standard nets), is cost-effective if it sustains high intervention coverage and prevents a resurgence in transmission [23]. Therefore, successful vector control depends on managing the long-term cross-sectoral threat through ‘One Health’ policies while simultaneously strengthening the immediate systems for net delivery, durability, and utilization.

This systematic review demonstrates the efficacy of chlorfenapyr-pyrethroid nets; however, several limitations qualify the generalizability and long-term applicability of these findings. The evidence is marked by substantial heterogeneity (I^2^ = 67%) in study design, vector species, and geography, limiting broad, uniform application of the findings. More critically, emerging data on long-term, programmatic durability present a significant caution. Results from a cluster-randomized trial in Benin show that while these nets provided strong protection in years 1 and 2, their epidemiological and entomological superiority was not sustained into the third year of community use [24]. This decline was linked to programmatic factors specifically, physical net deterioration and degrading insecticide concentration rather than to emerging metabolic resistance. Therefore, the operational lifespan of these nets for public health impact may be shorter than anticipated, dependent as much on physical durability as on initial insecticidal potency. Consequently, this synthesis, which draws heavily on controlled, shorter term efficacy studies, may overestimate the sustained protective effect of chlorfenapyr-pyrethroid nets under real-world program conditions over a standard 3-year distribution cycle.

Finally, the chosen methodological review, which focused on published English literature, risked selection bias due to non-English publications and unpublished reports. Also, the high statistical heterogeneity (I^2^ = 67%) observed across studies, despite the use of a random-effects model, indicates that the pooled effect estimate should be interpreted as an average of diverse contexts rather than a uniform effect.

## Conclusion

Chlorfenapyr-pyrethroid nets represent a significant advancement in the fight against pyrethroid-resistant malaria vectors. Their deployment is a necessary and effective short-to-medium-term strategy to reduce malaria transmission and prevent a resurgence of the disease. However, they are a perishable resource. The emerging signs of resistance signal that the window of utility may be narrow without decisive action. To sustain the gains, malaria control programs must adopt an integrated and proactive approach. This includes implementing chlorfenapyr nets as part of a rotation plan with other effective insecticide classes; enhanced surveillance through strengthening national systems for routine phenotypic and molecular resistance monitoring to enhance surveillance; adopting a ‘One Health’ framework to manage insecticide use across agriculture and public health; and investing in research on new insecticide chemistries, non-insecticidal tools, and refined resistance management strategies. It is further recommended that future research should focus on longitudinal studies of chlorfenapyr resistance dynamics, including mechanisms of resistance development and potential cross-resistance with other insecticide classes. There is also a need for operational and impact studies to evaluate the long-term effectiveness, durability, and cost-effectiveness of chlorfenapyr-based nets under routine programmatic conditions. Additionally, research should assess optimal insecticide rotation strategies and examine resistance spread across human, livestock, and agricultural interfaces to inform sustainable, One Health-aligned vector control.

However, to effectively oversee and coordinate these action-oriented recommendations, an organ such as a National Insecticide Resistance and Vector Control Coordination Committee (NIRVCCC) could be established or designated as the responsible monitoring organ. This body should be anchored within the Ministry of Health (e.g. through the National Malaria Control Programme) but operate as a multisectoral platform incorporating key institutions from health, agriculture, animal health, environment, and research sectors. Its core mandate may include the following: (i) guiding and monitoring insecticide rotation plans, including the deployment of chlorfenapyr-based nets; (ii) coordinating and standardizing phenotypic and molecular resistance surveillance through national reference laboratories and sentinel sites; (iii) operationalizing the ‘One Health’ framework by aligning policies and data across sectors; and (iv) prioritizing and tracking investment in research and innovation. Membership could include representatives from the Ministry of Health, Ministry of Agriculture, veterinary services, environmental authorities, national research institutions, and academia, ensuring accountability, policy coherence, and evidence-informed implementation.

The successful integration of these measures is paramount for protecting this critical intervention and achieving the ambitious targets of the Global Technical Strategy for Malaria by 2030.

## Supplementary Material

PRISMA Checklist Chlorfenapyr pyrethroid nets for pyrethroid resistant malaria vectors.docx

## Data Availability

All data generated or analyzed during this study are included in this published article. The source studies cited are publicly available in the referenced databases.
