# Supplementary material for: Chlorfenapyr-pyrethroid nets for pyrethroid-resistant malaria vectors: efficacy, resistance risks, and policy implications
Source: Glob Health Action. 2026 Mar 2;19(1):2629075. doi: 10.1080/16549716.2026.2629075 (PMC12954797; doi:10.1080/16549716.2026.2629075)
Supplement: PRISMA Checklist Chlorfenapyr pyrethroid nets for pyrethroid resistant malaria vectors.docx [file ZGHA_A_2629075_SM1578.docx]

**PRISMA 2020 Checklist**

**Systematic review on Chlorfenapyr–pyrethroid net efficacy and resistance in sub-Saharan Africa**

| Section & Topic | Item | Checklist requirement | Where reported | Page number in the manuscript |
| --- | --- | --- | --- | --- |
| TITLE | 1 | Type of the study identified as a systematic review. | Title & abstract | 1,2 |
| ABSTRACT | 2 | Provision of an unstructured summary but comprising background, objectives, methods, results, and conclusions. | Abstract | 1,2 |
| BACKGROUND/INTRODUCTION | | | | 2,3,4,5 |
| Rationale | 3 | Description of the rationale for the review in the context of existing knowledge. | Background | 3 |
| Objectives | 4 | Provide an explicit statement of the questions addressed. | Background | 4,5 |
| METHODS | | | | 5 |
| Eligibility criteria | 5 | Specify inclusion and exclusion criteria and how studies were grouped. | Methods: inclusion/exclusion criteria | 5 |
| Information sources | 6 | List all information sources (databases, registries, websites, and organizations). | Methods: search strategy | 5 |
| Search strategy | 7 | Provide full search strategies for all databases used. | Methods: search strategy | 5 |
| Selection process | 8 | State how studies were selected, including number of reviewers. | Methods: inclusion criteria | 5 |
| Data collection process | 9 | Describe methods used for data extraction. | Methods: Inclusion criteria | 5 |
| Data items | 10 | List all variables for which data were sought (e.g., mortality, resistance trends). | Methods: data items/primary review data | 5 |
| Study risk of bias assessment | 11 | Describe methods to assess risk of bias. | Not applicable / Not assessed | N/A |
| Effect measures | 12 | Specify effect measures used for outcomes (e.g., mortality rates). | Methods: data analysis | 5 |
| Synthesis methods | 13a | Describe methods for deciding study eligibility for synthesis. | Methods: synthesis | 5 |
|  | 13b | Describe methods for preparing data (e.g., handling missing data). | Methods: synthesis | 5 |
|  | 13c | Describe methods for tabulating results or presenting data visually. | Results Tables & Figures | 5 |
|  | 13d | Describe methods for synthesis (qualitative and quantitative). | Methods: random-effects meta-analysis; thematic synthesis | 5 |
|  | 13e | Describe methods for investigating heterogeneity. | Methods: random-effects meta-analysis; thematic synthesis | 5 |
|  | 13f | Describe sensitivity analyses, if conducted. | Not conducted | N/A |
| Reporting bias assessment | 14 | State methods used to assess risk of reporting bias. | Not conducted | N/A |
| Certainty assessment | 15 | Describe methods used to assess certainty (e.g., GRADE). | Not conducted | N/A |
| RESULTS | | | | 7.8.9.10 |
| Study selection | 16a | Describe search results, numbers screened, excluded, included. | Results: PRISMA Flow Diagram | 7 |
|  | 16b | Cite studies that appeared eligible but were excluded, with reasons. | Results | 7 |
| Study characteristics | 17 | Present characteristics of included studies. | Results: Table | 7,8 |
| Risk of bias in studies | 18 | Present risk of bias assessment for each study. | Not conducted | N/A |
| Results of individual studies | 19 | Present individual study results (effect estimates, data tables). | Results | 7,8 |
| Synthesis results | 20a | Summarize main findings for each synthesis. | Results | 9,10 |
|  | 20b | Present statistical synthesis results, including confidence intervals. | Results: meta-analysis | 7,8,9 |
|  | 20c | Explore heterogeneity. | Results | 7 |
|  | 20d | Sensitivity analyses, if conducted. | Not conducted | N/A |
| Reporting biases | 21 | Present reporting bias assessments. | Not applicable | N/A |
| Certainty of evidence | 22 | Present certainty assessments. | Not applicable | N/A |
| DISCUSSION | | | | 10,11 |
| Discussion | 23a | Provide a general interpretation of results. | Discussion | 10 |
|  | 23b | Discuss limitations of the evidence included. | Discussion | 11 |
|  | 23c | Discuss limitations of the review processes used. | Discussion | 11,12 |
|  | 23d | Discuss implications for policy, practice, and research. | Discussion | 11 |
| OTHER INFORMATION | | | | 13,15 |
| Registration and protocol | 24 | Provide registration details or state if unregistered. | Not registered | N/A |
| Support | 25 | Describe funding or support sources. | Acknowledgements | 13 |
| Competing interests | 26 | Declare conflicts of interest. | COI statement | 13 |
| Availability of materials | 27 | Provide data, code, and materials availability statement. | Data availability | 14,15 |
